# Information and Training on the Use of Telemedicine in Pediatric Population: Consensus Document of the Italian Society of Telemedicine (SIT), of the Italian Society of Preventive and Social Pediatrics (SIPPS), of the Italian Society of Pediatric Primary Care (SICuPP), of the Italian Federation of Pediatric Doctors (FIMP), and of the Syndicate of Family Pediatrician Doctors (SIMPeF)

**DOI:** 10.3390/jpm13020314

**Published:** 2023-02-11

**Authors:** Susanna Esposito, Cristiano Rosafio, Francesco Antodaro, Alberto Argentiero, Marta Bassi, Paolo Becherucci, Fabio Bonsanto, Andrea Cagliero, Giulia Cannata, Fabio Capello, Fabio Cardinale, Tiziana Chiriaco, Alessandro Consolaro, Angelica Dessì, Giuseppe Di Mauro, Valentina Fainardi, Vassilios Fanos, Alfredo Guarino, Giada Li Calzi, Elisa Lodi, Mohamad Maghnie, Luca Manfredini, Emanuela Malorgio, Nicola Minuto, Maria Grazia Modena, Rossano Montori, Andrea Moscatelli, Elisa Patrone, Elena Pescio, Marco Poeta, Angelo Ravelli, Maddalena Spelta, Agnese Suppiej, Sergio Vai, Luca Villa, Rinaldo Zanini, Renato Botti, Antonio Vittorino Gaddi

**Affiliations:** 1 Pediatric Clinic, Department of Medicine and Surgery, University of Parma, 43126 Parma, Italy; 2 Family Pediatrics, AUSL Modena, 41124 Modena, Italy; 3 Department of Pediatrics, IRCCS Giannina Gaslini, 16147 Genoa, Italy; 4 Family Pediatrics, 50139 Florence, Italy; 5 Alma Mater University, 40126 Bologna, Italy; 6 Family Pediatrics, 10121 Turin, Italy; 7 UO Territorial Pediatrics, Primary Care Department, AUSL Bologna, 40126 Bologna, Italy; 8 Department of Pediatrics and Emergency, Pediatric Hospital Giovanni XXIII, University of Bari, 70124 Bari, Italy; 9 Health Department, ASL Roma 3, 00125 Rome, Italy; 10 General Management, IRCCS Istituto Giannina Gaslini, 16147 Genoa, Italy; 11 Pediatric and Rheumatology Clinic, IRCCS Istituto Giannina Gaslini, 16147 Genoa, Italy; 12 Department of Neurosciences, Rehabilitation, Ophthalmology, Genetics and Maternal-Child Sciences (DINOGMI), University of Genoa,16126 Genoa, Italy; 13 Department of Surgical Sciences, University of Cagliari, 09127 Cagliari, Italy; 14 Family Pediatrics, 81100 Caserta, Italy; 15 Section of Pediatrics, Department of Translational Medical Sciences, University of Naples Federico II, 80131 Naples, Italy; 16 P.A.S.C.I.A. Center (Heart Failure Care Program, Childhood Heart Diseases and Those at Risk), University of Modena and Reggio Emilia, AOU Polyclinic of Modena, 41124 Modena, Italy; 17 Pediatric Pain and Palliative Care Service, IRCCS Istituto Giannina Gaslini, 16147 Genoa, Italy; 18 Community Medicine and Primary Care, AUSL Modena, 41124 Modena, Italy; 19 UOC Anesthesia and Intensive Care, IRCCS Istituto Giannina Gaslini, 16147 Genoa, Italy; 20 UOSID Trial Center, IRCCS Giannina Gaslini Institute, 16147 Genoa, Italy; 21 Scientific Directorate, IRCCS Istituto Giannina Gaslini, 16147 Genoa, Italy; 22 Pediatric Clinic, Department of Medical Sciences, University of Ferrara, 44124 Ferrara, Italy; 23 Center for Metabolic Diseases and Atherosclerosis, University of Bologna, 40126 Bologna, Italy

**Keywords:** telemedicine, teleconsultation, telepediatrics, telemonitoring, televisit

## Abstract

Telemedicine has entered the daily lives of doctors, although the digital skills of healthcare professionals still remain a goal to be achieved. For the purpose of a large-scale development of telemedicine, it is necessary to create trust in the services it can offer and to favor their acceptance by healthcare professionals and patients. In this context, information for the patient regarding the use of telemedicine, the benefits that can be derived from it, and the training of healthcare professionals and patients for the use of new technologies are fundamental aspects. This consensus document is a commentary that has the aim of defining the information on and training aspects of telemedicine for pediatric patients and their caregivers, as well as pediatricians and other health professionals who deal with minors. For the present and the future of digital healthcare, there is a need for a growth in the skills of professionals and a lifelong learning approach throughout the professional life. Therefore, information and training actions are important to guarantee the necessary professionalism and knowledge of the tools, as well as a good understanding of the interactive context in which they are used. Furthermore, medical skills can also be integrated with the skills of various professionals (engineers, physicists, statisticians, and mathematicians) to birth a new category of health professionals responsible for building new semiotics, identifying criteria for predictive models to be integrated into clinical practice, standardizing clinical and research databases, and defining the boundaries of social networks and new communication technologies within health services.

## 1. Introduction

While digital tools have been patchily established for several decades, the global public health challenges due to the COVID-19 pandemic catalyzed their use as an essential mechanism not only to strengthen emergency response, but also to ensure the continuity of health services [1,2,3]. In particular, the periods of limited mobility have underlined the need to resort to telemedicine, i.e., the provision of health-related services and information through information and communication technologies (ICTs), as a critical factor for expanding access to services and promoting continuity of care [4,5,6]. The technologies attribu1 overall to the sphere of telemedicine have clear benefits for the screening, diagnosis, management, treatment, and long-term follow-up even of pediatric subjects [7,8,9,10]. However, there are some barriers to the delivery of telehealth, including technology skills shortages and insufficient training of healthcare personnel and patients [11,12]. Therefore, strategic planning is necessary with the structural planning capabilities to transform global approaches to telemedicine into effective and widespread operating systems in all skills and expectations of professionals, as well as the broader population. Only in this way will it be possible to advance universal health coverage, protect the population in times of emergency, and improve health and wellbeing.

For the purpose of a large-scale development of telemedicine, it is necessary to create trust in the services it can offer and to favor their acceptance by healthcare professionals and patients [13,14]. In this context, information to the patient regarding the use of telemedicine, the benefits that can be derived from it, and the training of healthcare professionals and patients for the use of new technologies are fundamental aspects [1,15,16]. In fact, since it is a technological innovation, it is essential that healthcare professionals and patients are adequately trained and prepared, and that they aware of their role and of the effectiveness of the service, for the benefit of the health of the patients who benefit from it and the efficiency of the health system. Therefore, information and training actions are important to ensure the necessary professionalism and knowledge of the tools, as well as a good understanding of the contexts in which they are used. This consensus document is a commentary written by the main Italian scientific societies involved in the use of telemedicine in pediatrics. It has the aim of defining the information on and training aspects of telemedicine for pediatric patients and their caregivers, as well as pediatricians and other health professionals who deal with minors.

## 2. Methodology

The MEDLINE–PubMed database was searched from 2000 to 2022 to collect the literature. The following combination of keywords was used: “telemedicine” AND “information” OR “training” OR digital divide” AND “children” OR “paediatric” OR “pediatric” OR “adolescent”. We also performed a manual search of the reference lists of the obtained studies. Studies on telemedicine in chronic diseases and transition stages of life in childhood during COVID-19 pandemic were included.

## 3. Telemedicine, Training, and Technology

### 3.1. Reorganization of the Training Course

While it is true that skills need tools, the opposite is also true if they are to be effective. In fact, we are witnessing a profound reorganization of the healthcare system that makes it necessary to rethink the training of health professionals. Digitalization affects almost every aspect of modern daily life, including healthcare. The sustainability of introducing eHealth concepts based on information technology into clinical practice relies first and foremost on an effective rethinking of the training pathway. At present, there are degree programs whose curricula connect and integrate the traditional curriculum with branches and sectors such as biomechanics, biotechnology, computer science, and the digital domain. With regard to artificial intelligence (AI), Italian universities offer more than 200 curricula in AI spread over about 50 universities; in 2021, Italy launched the national doctorate in artificial intelligence (PhD-AI.it) [1]. These curricula can be complemented by level I or II master’s degrees on telemedicine, digital medicine, AI, and eHealth management.

Thus, the issue of digital competencies in healthcare arises, which is not only a question of health service management. The skills required for the healthcare professional of the near future are diverse and do not necessarily coexist in the same person. The contributions of telemedicine have nothing to do with technology. They are a matter of accepting a change in mentality. From the self-sufficiency of skills, we have to move toward a model characterized by a decentralization of skills across different subjects called upon to share the capacity to integrate their contribution in an interoperable project process. Any decoupling mechanism must, therefore, be avoided, i.e., the introduction of technologies, platforms or processes disconnected from the appropriate and necessary training of users and users. For this reason, it is necessary that the development of guidelines and digital tools integrate “by design” sections referring to training.

The need to reorganize training in an integrated and homogeneous manner is not only necessary but obligatory. Only through a path of gradual and structural acquisition of integrated competences is it possible to resolve doubts concerning the doctor–patient relationship, data security and privacy, and questions of accountability and reliability of information, as well as bridge the gap between current and future health professionals who differ in their perceptions of eHealth and telemedicine [17].

### 3.2. Strategies to Reduce the “Digital Divide” in Operators and Users

Technological innovation and the digital transition on which telemedicine is based require reducing the present digital divide among Italian citizens, favoring education in future technologies and maximum digital inclusion. The “Italy 2025 Strategy” promoted by the Prime Minister’s Office, which aims to make digital public services inclusive and accessible to all, falls within this framework [18]. Recent data have shown that 58% of Italians aged between 16 and 74 (26 million citizens) lack basic digital skills (the EU average is 42%) [19]. Of these, 11 million are not Internet users. The lack of technological knowledge produces multiple effects and has very significant economic and social consequences [20,21].

An operational plan to bridge the digital divide sees actions on four axes: education, workforce, ICT skills, and citizens [22]. The gap, however, is not only cultural but also structural. For the educational and profound change initiatives outlined above to have a chance of success, the barriers of access to quality connectivity with guaranteed minimum performance must be removed. The strength of a telemedicine project also lies in ensuring that, for example, the telemonitoring flow of a frail patient is not slowed down or interrupted. It is, therefore, necessary to include secondary actions and contingency operational plans in the design phase that safeguard the flow of data and secure the patient’s health.

In the health sector, digital divide reduction strategies should be adapted to consider the complexity and specificity of each context [23]. Health professionals will be under greater pressure to change and will also be called upon to facilitate this transformation in their patients. For this very reason, it seems useful and urgent to plan specific and well-designed training initiatives to provide practical tools that can support this change.

### 3.3. Certification of Competences and Training Courses

The COVID 19 pandemic—like any period characterized by highly critical situations—induced profound changes in the daily practices of people and organizations [24]. Some changes such as telemedicine, developed to reduce contact and, thus, contagion between users and professionals, have shown a positive innovative potential even outside strictly pandemic times [25]. Like any new practice, especially if highly innovative, it is easy to run into organizational and implementation interpretations with such a degree of diversification that many of the potential benefits are nullified. In the case of the “telemedicine system” this risk appears particularly evident because many of the potential benefits, even outside pandemic times, are achievable precisely with large-scale applications capable of connecting many tens of thousands of users with multiple professionals in different organizations [26].

Telemedicine requires, like most care practices but to a greater than average extent, shared operational applications, replicable with interchangeable contents and capable of being read and interpreted by multiple professionals—even simultaneously—operating in different and distant organizations [27]. These indispensable characteristics can be achieved by means of a systemic organizational process tailored to the different needs of the organizations but transversal to them, and they can be maintained over time by means of a training process proposed to all the professionals involved, capable of certifying the skills of the various roles—project, programming, and utilization—necessary for the proper functioning of the complex system on which telemedicine is based [28].

Such articulated, complex training systems, addressed to many healthcare operators and on which a specific historical experience is lacking, increase their effectiveness and efficiency if they are based on certification models capable, perhaps with cascade models that start from national indications with guidelines and then be declined in a specific way at a local level, of responding in a flexible but univocal way to the real knowledge needs of the operators involved [29]. The scope that telemedicine represents as a tool for scientific research should also be emphasized. Certifying the competences by certifying the training paths appears to be the main way to make the most of telemedicine for users, professionals, and organizations in terms of clinical and organizational scientific research [29].

### 3.4. Parental Training

The organizational proposal that arises using telemedicine is not simply a new technical modality to address a clinical problem. Telemedicine represents a completely new modality from a clinical, organizational, cultural, experiential, and medico-legal point of view. Each of these areas must be addressed by both professionals and users.

Outside the period of the emergency caused by the onset of the pandemic, if the potential benefits of telemedicine (such as facilitated access to health information and services for the persons concerned and at the appropriate time) are to be fully utilized, the active involvement of users becomes indispensable [25,26]. Training becomes the tool with which to pursue this important step. Our care culture is based on the therapeutic alliance between users and professionals. Many of the results (e.g., screening, preventive medicine, sharing of lifestyles, and compliance with therapy) achieved today are based on this alliance [30]. Without a specific and extensive training activity, addressed to users on the language, values, technical value, and tools used by telemedicine, it becomes difficult to imagine that it is possible to maintain a high level of therapeutic alliance outside the pressure of emergency situations.

Telemedicine cannot completely replace “presence and contact medicine”. Undoubtedly, however, it can be a tool that improves many services compared to the traditional approach. Health services that are more easily, efficiently, and rapidly accessible through telemedicine can contribute to good outcomes and help move away from reactive to proactive approaches to preserving health [31]. The rapid and virtual breakdown of the space/time dimension of telemedicine allows professionals to interact more frequently with patients and their families, who can, in turn, be more easily involved and increase their experience of active therapeutic alliance [25,26]. For this to really happen, it is inconceivable that training activities for users tailored to their abilities and interests should not be planned: specific training for different age groups for the pediatric population and for families [25].

Already today, it happens with great frequency that users use the main search engines in parallel with contact with the practitioner. Telemedicine cannot be confused with a Google approach or, even worse, as a system aimed solely at saving time and economic resources. Telemedicine is not just an innovative language but represents a different culture of the caregiver/user relationship. It is not enough for users to learn a new operating mode or a new procedure; they need to take possession of a new culture, perceiving its advantages and limitations. For this to happen, healthcare organizations must take charge of training processes [31]. If, in the near future, they are to make the best use of the advantages of telemedicine while avoiding its risks, a training campaign directed at users and shared with health professionals must be devised.

In order for a relationship of trust mediated by “distance medicine” at least equal to that generated by “in-presence medicine” to arise even outside emergency situations, it is necessary to define and disseminate between health professionals and users the elements that can give rise to an empathic relationship even at a distance [25,26]. If, on the one hand, digital technologies have facilitated connections between individuals that were unthinkable only a few years ago, on the other hand, they run the risk of a deterioration of interpersonal relationships. The development of caregiver/user communication skills, therefore, becomes necessary and must be emphasized in shared educational processes [32,33]. These paths are not easy, but they are indispensable and, although long, must begin as soon as possible in conjunction with the implementation of the telemedicine organizational model.

### 3.5. The Experience of the IRCCS Giannina Gaslini in Genoa, Italy

The IRCCS Gaslini in Genoa, Italy (which encompasses the mission of care and research) and the role of reference center for pediatric medicine have made the option of telemedicine a nodal point for research, care, and a pillar for the development and growth of the institute itself. The institute, despite still uncertain resources and scenarios (e.g., the fund for inter-regional telemedicine projects has not yet been allocated to the regions), has started with a bottom-up approach (systematically involving all its resources) and with a clinical and research approach oriented toward social innovation and aimed at enhancing the work previously carried out; the first and initial manuals and use cases have, therefore, been defined, coordinated, and correlated with what is being developed at a regional level. This activity, conducted internally with a strategic project management approach, was initiated by identifying, in addition to internal resources, a coordination and liaison function headed by a social innovation manager, a telemedicine expert physician, and a process expert physician, who activate and foster moments of collaboration among all participants (through the exchange of each document among themselves and with others, thus facilitating the circularity not only of information but also of the different points of view/needs).

We began by first of all questioning ourselves on a “method” to support the introduction of telemedicine, trying to define the prerequisites for an enabling context and, at the same time, able to generate a shared vision. This is in order to overcome a tendency to identify “departmental” solutions and, on the contrary, to support a logic of organizational learning for the entire system so that, in the case of telemedicine, every time an operating unit asks to start up an activity, it remains possible to detect specificities to be reconciled and/or procedures to be reviewed right from the initial stages. The actions promoted by the Gaslini reality were, therefore, conceived starting from a few simple methodological rules:

- Informal identification of micro-communities of practice (multiple professional working groups involved in telemedicine services);
- Progressive involvement of the various corporate functions concerned as the procedure defining purposes, activities, and responsibilities takes shape;
- Autonomy and responsibility (accountability) of the functions involved from time to time (also of the second line, not only the top management) ensuring, in addition to a top-down direction of the communication of strategic objectives, a circularity in the method of gathering and dissemination of information, and proposals stimulated with a bottom-up logic.

The approach may appear time-consuming (or slow). The aim, however, is not simply to introduce a technology/innovation, but to reach out and listen in a cascade and almost capillary manner to the institute’s various professionals in order to create the conditions for a cultural change that will lead to valuing and using technology appropriately, giving time to break in internal steps and procedures that fall under the responsibility of different offices that seize the opportunity to review and update them, in order to improve the functioning of the overall system.

This method of involvement has generated a change in the initial climate, which can be seen both through the increase in the number of spontaneous and noninduced requests by the operating units to start telemedicine services, and through the elaboration and proposal by the various offices involved of improved solutions to make the individual steps of an organizational process increasingly smooth. This organizational process is encapsulated in two other expressions that are reported here, with value as a thermometer with respect to the method: (1) with respect to “before”, we are put in a position to understand to where and why and, therefore, to contribute to the how; (2) realizing that, instead of having the responsibility to do it oneself, also having the responsibility to not take for granted what it takes to do and complete it better and sooner.

Each of the activities described above is developed with attention to the following underlying issues:

- Definition of procedures shared by management and professionals to ensure unity of supply, safety, and appropriateness with a view to the growth of local clinical networks and with a view to growth at an extra-regional level;
- Reflection on the continuous use of data for the improvement of diagnosis, therapy, rehabilitation and care;
- Definition and/or refinement of the rules of engagement (e.g., pricing, pricing, minimum technological, and organizational requirements for the accreditation of the various services) necessary to ensure inter-regionality (at present, for example, only television is priced) and the ability to also compete with a private offer;
- Privacy by design so as to anticipate the privacy issue from the outset, identifying solutions upstream and with a system view;
- In prospect, to extend participation in the communities of practice for telemedicine services to the referents of the decentralized workplaces acquired with the “widespread Gaslini”, an organizational model desired by the region for which, as from 1 July 2022, the Pediatrics and Neonatology Units of the Ligurian Local Health Authorities have become “company branches” of Gaslini from a hub and spoke perspective.

## 4. Conclusions

Digital skills are a key theme of innovation in the national health service, for all the players involved: patients, clinicians, technicians, IT specialists, and managers. Undoubtedly, as in any other path concerning health and care, training tools are at the heart of innovation. For the present and the future of digital healthcare, there is a need for a growth in the skills of professionals and a lifelong learning approach throughout one’s professional life. Therefore, information and training actions are important to guarantee the necessary professionalism and knowledge of the tools, as well as a good understanding of the interactive context in which they are used.

Patients assisted using telemedicine systems and their caregivers require training that must not be limited to the technological aspects, but also intervene on the social and relationship aspects, on the change in the doctor–patient relationship, and on the reassurance that, even at a distance, assistance with and care for the patient and their pathology are still guaranteed. Furthermore, for the purpose of a wide diffusion of telemedicine, particular attention must be paid to the training and updating of health professionals, to familiarize them with the new methods for the exercise of their profession. Training should cover new information acquisition equipment and data transmission technologies. Furthermore, psychological training will also be essential in order to humanize the remote relationship and to remedy the lack of that physical presence on which the dialogue between doctor and patient has so far been based.

It is crucial that learning becomes a systemic action and not an extemporaneous proposal. It is, in fact, essential to implement a structured university training program, combined with service training, aimed at optimizing the use of telemedicine to improve the quality of assistance. Furthermore, medical skills can also be integrated with the skills of various professionals (engineers, physicists, statisticians, and mathematicians) to birth a new category of health professionals responsible for building new semiotics, identifying criteria for predictive models to be integrated into clinical practice, standardizing clinical and research databases, and defining the boundaries of social networks and new communication technologies within health services.

## Data Availability

Not applicable.
